# Coronary vasculature patterning requires a novel endothelial ErbB2 holoreceptor

**DOI:** 10.1038/ncomms12038

**Published:** 2016-06-30

**Authors:** Haig Aghajanian, Young Kuk Cho, Lauren J. Manderfield, Madison R. Herling, Mudit Gupta, Vivienne C. Ho, Li Li, Karl Degenhardt, Alla Aharonov, Eldad Tzahor, Jonathan A. Epstein

**Affiliations:** 1 Department of Cell and Developmental Biology, Penn Cardiovascular Institute, Institute for Regenerative Medicine, Perelman School of Medicine at the University of Pennsylvania, Philadelphia, Pennsylvania 19104, USA; 2 Department of Pediatrics, Chonnam National University Medical School, Gwangju 61186, South Korea; 3 Department of Pediatrics, Children's Hospital of Philadelphia, University of Pennsylvania, Philadelphia, Pennsylvania 19104, USA; 4 Department of Biological Regulation, Weizmann Institute of Science, Rehovot 76100, Israel

## Abstract

Organogenesis and regeneration require coordination of cellular proliferation, regulated in part by secreted growth factors and cognate receptors, with tissue nutrient supply provided by expansion and patterning of blood vessels. Here we reveal unexpected combinatorial integration of a growth factor co-receptor with a heterodimeric partner and ligand known to regulate angiogenesis and vascular patterning. We show that ErbB2, which can mediate epidermal growth factor (EGF) and neuregulin signalling in multiple tissues, is unexpectedly expressed by endothelial cells where it partners with neuropilin 1 (Nrp1) to form a functional receptor for the vascular guidance molecule semaphorin 3d (Sema3d). Loss of Sema3d leads to improper patterning of the coronary veins, a phenotype recapitulated by endothelial loss of ErbB2. These findings have implications for possible cardiovascular side-effects of anti-ErbB2 therapies commonly used for cancer, and provide an example of integration at the molecular level of pathways involved in tissue growth and vascular patterning.

## Abstract (web summary)

Semaphorin ligands and cognate receptors are important in patterning the vasculature. Here, Aghajanian *et al.* report an unexpected role for ErbB2 in endothelial cells where it partners with Nrp1 to form a novel semaphoring holoreceptor required for embryonic vascular patterning.

## Graphical abstract

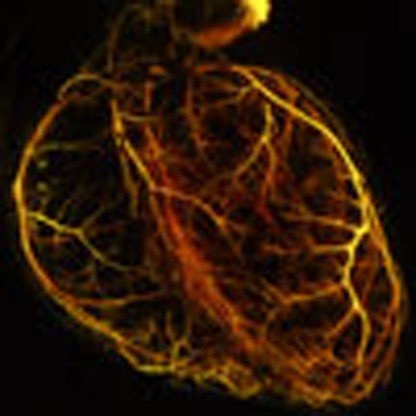

Early in mammalian development, circulating blood within the heart chambers is sufficient to provide oxygen and nutrients to the surrounding myocardium through simple diffusion. As the embryo grows, increased demand is placed on the heart to pump blood to the body, and the myocardium thickens. The coronary vasculature develops to supply myocardial cells that can no longer be nutritionally sustained by simple diffusion from the cardiac lumen. Congenital abnormalities in coronary development can lead to pathologies such as myocardial ischaemia, malignant arrhythmias and sudden death^1^.

Until the late 1980s, it was believed that the coronary arteries and veins arose through angiogenesis from their points of origin, the aorta and coronary sinus, respectively^2^. That notion was challenged when it was shown that the coronary vasculature is present in the heart well before there are connections to the circulation^3^. Recently, a number of studies have identified various sources of the endothelial cells that give rise to the coronary arterial and venous vasculature including the proepicardium^4^, epicardium, endocardium and sinus venosus^5^,^6^. Cells migrating from these sources form tubular structures and a primary vascular plexus surrounding the heart. During this time, the arterial vascular tubes around the aortic trunk penetrate the aorta, while the venous capillary beds form connections with the coronary sinus allowing for circulation of blood through the coronary vessels. There are some clues to the cellular and molecular mechanisms required for coronary arterial connection to the aorta^7^,^8^. However, very little, if anything, is known about the mechanisms mediating coronary venous connections.

Class 3 semaphorins (Sema3) compose a family of secreted ligands that have been shown to be important guidance molecules in cardiovascular development and vascular patterning^9^,^10^,^11^,^12^ in addition to their role in guiding neuronal path-finding^13^. Although Sema3e can bind to and signal via plexin D1 alone^12^, most Sema3 proteins require the co-receptor neuropilin 1 (Nrp1) or neuropilin 2 to bind in conjunction with a signalling receptor such as a plexin^14^. Neuropilins are also known to heterodimerize with various related tyrosine kinase receptors such as Vegfr2 (KDR)^15^.

Erbb2 receptor tyrosine kinase 2 (ErbB2) is a member of the epidermal growth factor receptor (EGFR) family. ErbB2 is unique among EGFR in that it has no known direct ligand and generally functions as a co-receptor^16^. Although ErbB2 expression is well characterized in myocardium^17^ and plays a critical role in cardiomyocyte proliferation and differentiation during development^18^,^19^, a role in endothelial function has yet to be described.

Here we demonstrate that ErbB2 interacts with Nrp1 to form a Sema3d co-receptor expressed by developing coronary endothelium and required for proper connections of the forming coronary veins to the right atrium. Genetic deletion of Sema3d or endothelial ErbB2 results in similar pathological venous phenotypes. Thus, ErbB2 plays an unexpected and important role in the vasculature.

## Results

### Abnormal coronary connections in Sema3d nulls

**Figure 1:**
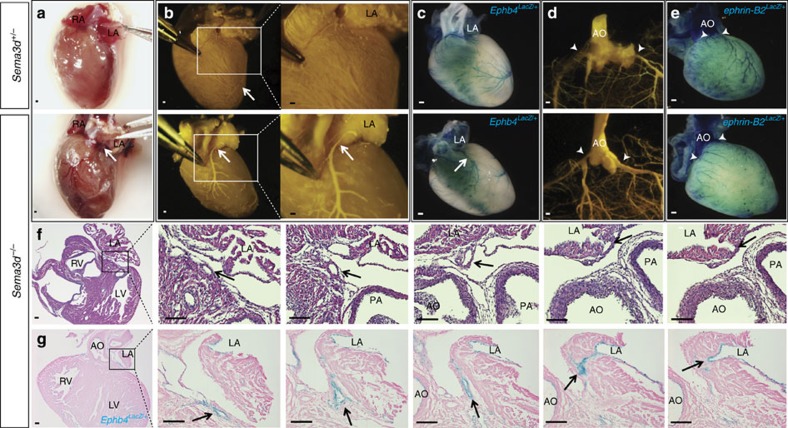
Coronary veins abnormally connect to the left atrium in *Sema3d*^*−*/*−*^ hearts. (**a**) Left anterior oblique view of adult *Sema3d*^*+/−*^ (top) and *Sema3d*^*−/−*^ (bottom) hearts. The anterior interventricular vein (arrow) can be seen connecting to the left atrium in the *Sema3d*^*−/−*^ heart. (**b**) Mircrofil perfusion of 3 week old hearts from *Sema3d*^*+/−*^ (top) and *Sema3d*^*−*/*−*^ (bottom) mice. (**c**) Whole-mount X-gal staining of *Sema3d*^*+/−*^*;Ephb4*^*LacZ/+*^ (top) and *Sema3d*^*−/−*^*;Ephb4*^*LacZ/+*^ (bottom) postnatal hearts. (**d**) Cleared *Sema3d*^*+/−*^ (top) and *Sema3d*^*−/−*^ (bottom) postnatal hearts perfused with Microfil to visualize the origin of the coronary arteries (arrowheads). (**e**) Whole-mount X-gal staining of *Sema3d*^*+/−*^*;ephrin-B2*^*LacZ/+*^ (top) and *Sema3d*^*−/−*^*;ephrin-B2*^*LacZ/+*^ (bottom) postnatal hearts verifying arterial identity of the coronary vessels originating from the aorta (arrowheads). (**f**) Photomicrographs of H&E stained frontal sections from a *Sema3d*^*−/−*^ heart following a coronary vein (arrows) from the left anterior AV groove connecting with the left atrial lumen. (**g**) Photomicrographs of eosin and X-gal stained frontal sections from a postnatal *Sema3d*^*−/−*^*;Ephb4*^*LacZ/+*^ heart following a coronary vein (arrows) from the left anterior AV groove connecting with the left atrial lumen. AO, aorta; LA, left atrium; LV, left ventricle; PA, pulmonary artery; RV, right ventricle. Scale bars, 100 μm.

We examined the hearts of postnatal *Sema3d*^*−/−*^ mice to investigate the effects of Sema3d on the development of the coronary vasculature. Unlike human, murine coronary vessels reside in two distinct anatomical compartments. The coronary veins are subepicardial and are visible on the heart surface, while the coronary arteries are intramyocardial and cannot be visualized on gross inspection. Surface vessels, the presumptive coronary veins, are seen abnormally connecting to the left atrium in *Sema3d*^*−/−*^ adult hearts, but not in *Sema3d*^*+/−*^ controls (Fig. 1a). To better visualize the coronary vessels and connections, we perfused the vasculature with a low viscosity compound (Microfil) and observed that ∼30% of the *Sema3d*^*−/−*^ hearts (8/27) displayed abnormal connections of the anterior interventricular vein to the left atrium, while none (0/36) of the *Sema3d*^*+/−*^ controls exhibited this abnormal connection (Fig. 1b).

To verify the arterial/venous identities of the coronary vessels, we crossed the *Sema3d*^*+/−*^ mice with either an arterial (*ephrin-B2*^*LacZ*^) or venous (*Ephb4*^*LacZ*^) endothelial reporter. Whole-mount X-gal staining of *Sema3d*^*−/−*^*;Ephb4*^*LacZ/+*^ hearts shows *Ephb4*-positive vessels abnormally connecting to the left atrium (Fig. 1c, arrow). These connections are absent in the *Sema3d*^*+/−*^*;Ephb4*^*LacZ/+*^ controls (Fig. 1c). Arterial Microfil perfusion reveals normal origins of the coronary arteries in both *Sema3d* null and control hearts (Fig. 1d). X-gal staining of *Sema3d*^*+/−*^*;ephrin-B2*^*LacZ/+*^ and *Sema3d*^*−/−*^*;ephrin-B2*^*LacZ/+*^ hearts confirms the arterial identity of these coronary vessels (Fig. 1e). These data suggest that Sema3d specifically affects the connections of the coronary veins and not coronary arteries.

We examined hearts from a developmental series of *Sema3d*^*−/−*^*; Ephb4*^*LacZ/+*^ and *Sema3d*^*−/−*^*;ephrin-B2*^*LacZ/+*^ embryos, and litter mate controls, to determine if the initial formation of coronary vessels was perturbed. These experiments indicate that the emergence of *Ephb4+* venous coronary endothelium and assembly of the primitive vascular plexus that matures into the coronary venous system is unaffected by the absence of Sema3d, and the first reproducible defect that we observed was an abnormal connection to the left atrium evident by E15.5 (Supplementary Fig. 1). No clear abnormalities of arterial endothelial specification, plexus formation or connections with the aorta were identified (Supplementary Fig. 2).

To further examine the nature of the abnormal venous connections, we examined serial hematoxylin and eosin stained sections, which confirmed the existence of an abnormal coronary vascular connection to the left atrium in *Sema3d*^*−/−*^ hearts. The abnormal vessel is superficial, consistent with venous identity, and traverses the left anterior atrioventricular groove before emptying into the left atrial lumen (Fig. 1f). X-gal and eosin stained serial sections from a *Sema3d*^*−/−*^*;Ephb4*^*LacZ/+*^ heart confirm the venous identity of this mispatterned vessel (Fig. 1g).

### Sema3d restricts coronary endothelium

**Figure 2:**
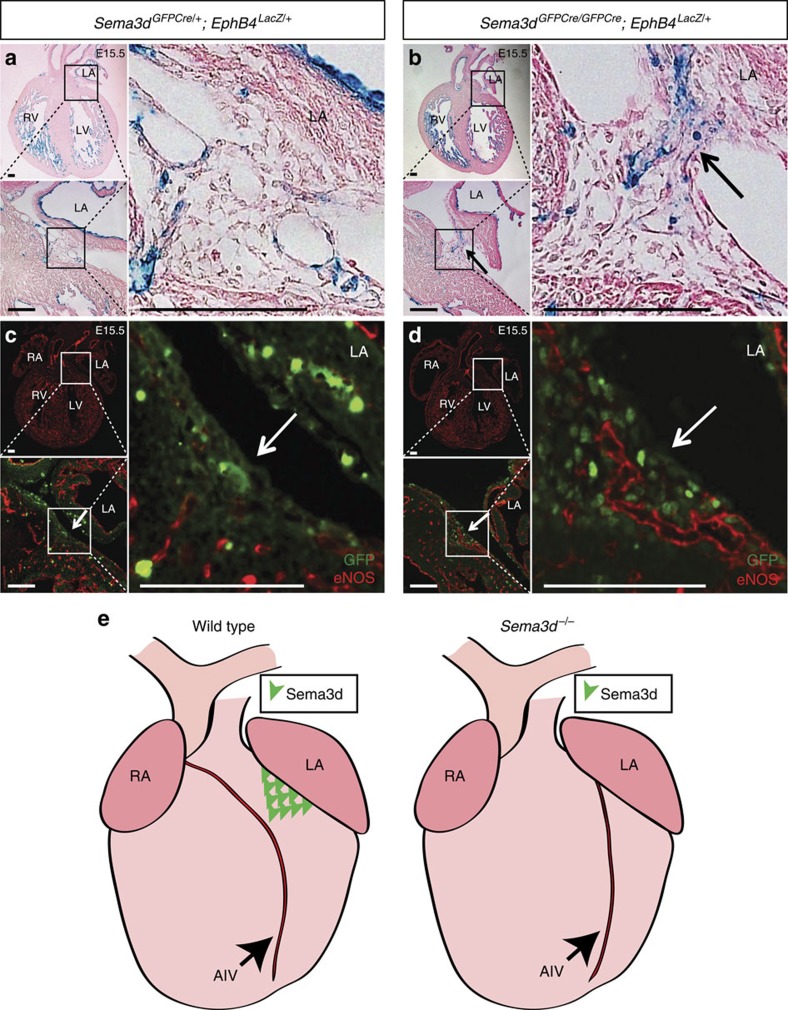
*Sema3d* expression excludes endothelium from the AV groove. (**a**,**b**) X-gal and eosin staining of *Sema3d*^*GFPCre/+*^*;Ephb4*^*LacZ/+*^ (**a**) and *Sema3d*^*GFPCre/GFPCre*^*;Ephb4*^*LacZ/+*^ (**b**) E15.5 heart frontal sections. Numerous LacZ positive (venous) cells can be observed in the left anterior AV junction of the *Sema3d* nulls (**b**; arrow) but not in the control (**a**). (**c**,**d**) Immunofluorescence for the endothelial marker eNOS (red) and GFP (green) of E15.5 *Sema3d*^*GFPCre/+*^*;Ephb4*^*LacZ/+*^ (**c**) and *Sema3d*^*GFPCre/GFPCre*^*;Ephb4*^*LacZ/+*^ (**d**) heart frontal sections. The GFP expressing left anterior AV groove (arrow) is mostly avascular in the control (**c**) but penetrated by numerous eNOS-positive (red) vessels in the *Sema3d* null (**d**). (**e**) Model depicting the role of Sema3d in coronary venous connection. In wild type mice, Sema3d is expressed in the left anterior AV groove and repels venous endothelium from abnormally forming connections with the left atrium (left), but in the absence of Sema3d the coronary anterior interventricular vein (AIV) abnormally connects to the LV (right). LA, left atrium; LV, left ventricle; RA, right atrium; RV, right ventricle. Scale bars, 100 μm.

We further examined venous endothelial patterning at E15.5 when vascular defects were first detected in the *Sema3d* null hearts. X-gal staining of E15.5 *Sema3d*^*+/−*^*;Ephb4*^*LacZ/+*^ heart sections reveals little to no venous endothelium in the left anterior atrioventricular groove (Fig. 2a). This is in contrast to numerous LacZ-positive venous endothelial cells seen traversing this junction and in close association with the left atrium in *Sema3d*^*−/−*^*;Ephb4*^*LacZ/+*^ embryonic hearts (Fig. 2b). Examination of *Sema3d*^*GFPCre/+*^ hearts, which have green fluorescent protein (*GFP*) knocked into the *Sema3d* locus resulting in a null allele^4^,^11^, reveals that *Sema3d* is expressed within the left anterior atrioventricular groove (Fig. 2c, arrow). In control embryos, this area of *Sema3d* expression is relatively avascular compared with the surrounding myocardium as determined by a lack of endothelial nitric oxide synthase (eNOS) expression (Fig. 2c). GFP is similarly expressed in the left anterior atrioventricular groove of the *Sema3d*^*GFPCre/GFPCre*^ null hearts (Fig. 2d, arrow), and lineage-tracing *Sema3d*-expressing cells gives rise to a similar pattern of cells populating the atrioventricular groove in *Sema3d* null and control hearts (Supplementary Fig. 3). But unlike the controls, numerous eNOS-positive vessels are seen penetrating this previously avascular zone in *Sema3d*^*GFPCre/GFPCre*^ hearts (Fig. 2d). Given the ability of Sema3d to function as an endothelial repellent^20^, we interpret these findings to suggest that Sema3d forms a barrier to restrict venous endothelial cells from populating the left anterior atrioventricular groove thereby preventing aberrant connections with the left atrium (Fig. 2e).

### ErbB2 is a co-receptor for Sema3d

**Figure 3:**
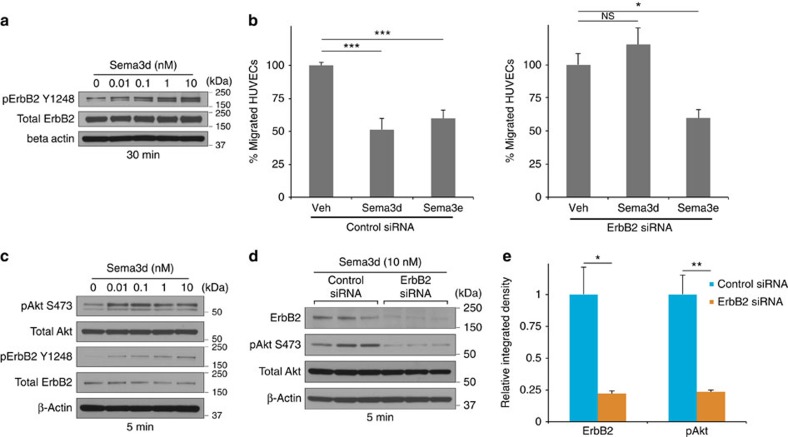
Sema3d signals via ErbB2 in endothelial cells. (**a**) Western blot for phospho-ErbB2 (Y1248) from HUVECs incubated with increasing concentrations of Sema3d for 30 min. (**b**) Transwell migration assay showing the percentage HUVECs that migrated through a porous membrane in the presence of Sema3d or Sema3e when compared to vehicle control in the presence of a control siRNA (left) or ErbB2 siRNA (right). ***P<0.001, *P<0.05, NS, not significant (one-way ANOVA between groups *P*<0.001 for both groups; *post-hoc* multiple comparisons, Tukey's test) (**c**) Western blot for phospho-Akt (S473) and phospho-ErbB2 (Y1248) from HUVECs incubated with increasing concentrations of Sema3d for 5 min. (**d**) Western blot for ErbB2 and phospho-Akt (S473) of HUVECs in the presence of control or ErbB2 siRNA treated with Sema3d (10 nM) for 5 min. (**e**) Quantification of ErbB2 knockdown and Akt phosphorylation from (**d**). **P<0.01, *P<0.05 (Student's *t*-test, *n*=3).

Sema3d binds to and requires Nrp1 for signalling to endothelial cells^11^,^20^, but a signalling co-receptor for Sema3d has yet to be fully described. To discover the endothelial signalling co-receptor for Sema3d, we conducted a receptor tyrosine kinase screen (see Methods section) and identified ErbB2 as a potential Sema3d co-receptor. ErbB2 is phosphorylated in response to Sema3d in human umbilical vein endothelial cells (HUVECs) in a dose-dependent manner (Fig. 3a). To determine the requirement of ErbB2 in Sema3d-mediated endothelial guidance, we employed a transwell migration assay. Both Sema3d and Sema3e are able to inhibit the migration of endothelial cells through a porous membrane (Fig. 3b). Knockdown of ErbB2 specifically abrogates the ability of Sema3d, but not Sema3e, to inhibit endothelial migration (Fig. 3b). We previously demonstrated Sema3d-dependent phosphorylation of Akt and the requirement of PI3K/Akt signalling in Sema3d-mediated endothelial guidance^20^. Akt and ErbB2 phosphorylation in response to Sema3d are both evident within 5 min of incubation (Fig. 3c). Small interfering RNA (siRNA) knockdown of ErbB2 in HUVECs abrogates Sema3d-induced Akt phosphorylation (Fig. 3d,e), indicating that ErbB2 is necessary for downstream repellent signalling mediated by Sema3d in HUVECs.

### ErbB2 in coronary endothelium

**Figure 4:**
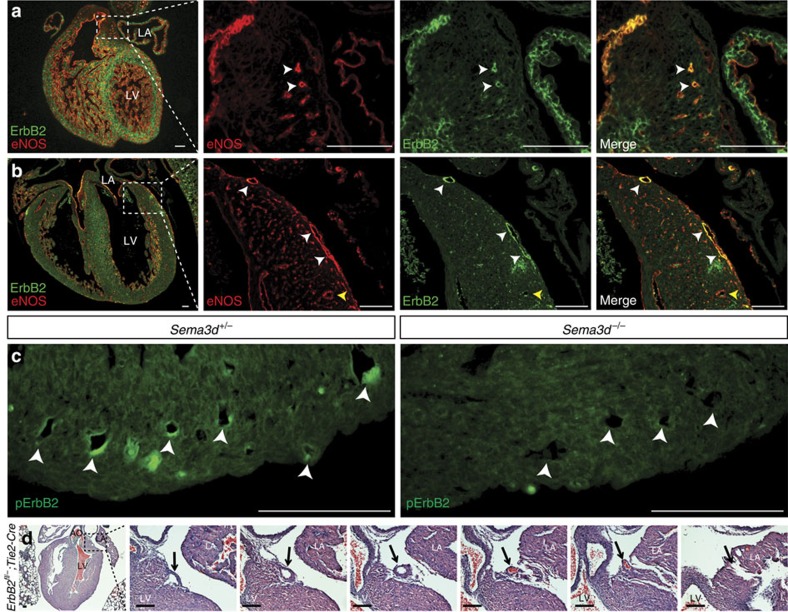
ErbB2 is expressed by coronary venous endothelium. (**a**) Immunofluorescence of a frontal section of an E14.5 heart for the endothelial marker eNOS (red) and ErbB2 (green). ErbB2 and eNOS co-localize to vessels in the antrioventricular junction (arrowheads). (**b**) Frontal section of an E17.5 heart co-stained for eNOS (red) and ErbB2 (green) demonstrating co-localization in subepicardial vessels (white arrowheads) but not in intramyocardial vessels (yellow arrowheads). (**c**) Phospho-ErbB2 (Y1248) immunofluorescence from frontal sections of E14.5 *Sema3d*^*+/−*^ and *Sema3d*^*−/−*^ hearts. ErbB2 phosphorylation is reduced in the *Sema3d*^*−/−*^ hearts (arrowheads). (**d**) Photomicrographs of H&E stained frontal sections from a postnatal *ErbB2*^*fl/−*^*;Tie2-Cre* heart tracing a vessel from the left anterior AV groove to the left atrial lumen (arrows). LA, left atrium; LV, left ventricle. Scale bars, 100 μm.

If ErbB2 is directly participating in Sema3d-mediated receptor signalling during coronary vein development, we predicted that it would be expressed by nascent coronary venous endothelium just before the time when abnormal venous connections are seen in *Sema3d* mutant embryos. At E14.5, shortly before anomalous venous connections are observed in Sema3d null hearts, eNOS-positive endothelial tubes co-express ErbB2 (Fig. 4a). Myocardial ErbB2 expression is also evident, consistent with previous observations of ErbB2 expression by cardiac muscle at earlier embryonic time-points^21^ (Fig. 4a). Expression of ErbB2 by coronary venous endothelium persists through at least E17.5 and is largely restricted in the heart to subepicardial (venous) endothelial cells (Fig. 4b, Supplementary Fig. 4, white arrowheads) and not intramyocardial (arterial) coronary vessels (Fig. 4b, Supplementary Fig. 4, yellow arrowheads). At E14.5, Y1248 phosphorylation of ErbB2 is detected by immunofluorescence in coronary endothelium of *Sema3d*^+/*−*^ hearts, consistent with receptor activation (Fig. 4c). However, phospho-ErbB2 is not detected in coronary endothelium of *Sema3d*^*−/−*^ hearts (Fig. 4c). Importantly, genetic deletion of ErbB2 in endothelium during development, using *Tie2-Cre*, recapitulates the anomalous pulmonary venous connection phenotype previously described in *Sema3d*^*−/−*^ mice^11^(Supplementary Fig. 5, Supplementary Video). Moreover, endothelial deletion of ErbB2 results in the anomalous coronary venous connection to the left atrium described here in *Sema3d*^*−/−*^ mice (Fig. 4d), providing strong genetic evidence for ErbB2 functioning in a signalling pathway with Sema3d.

### ErbB2 and Nrp1 form a Sema3d holoreceptor

**Figure 5:**
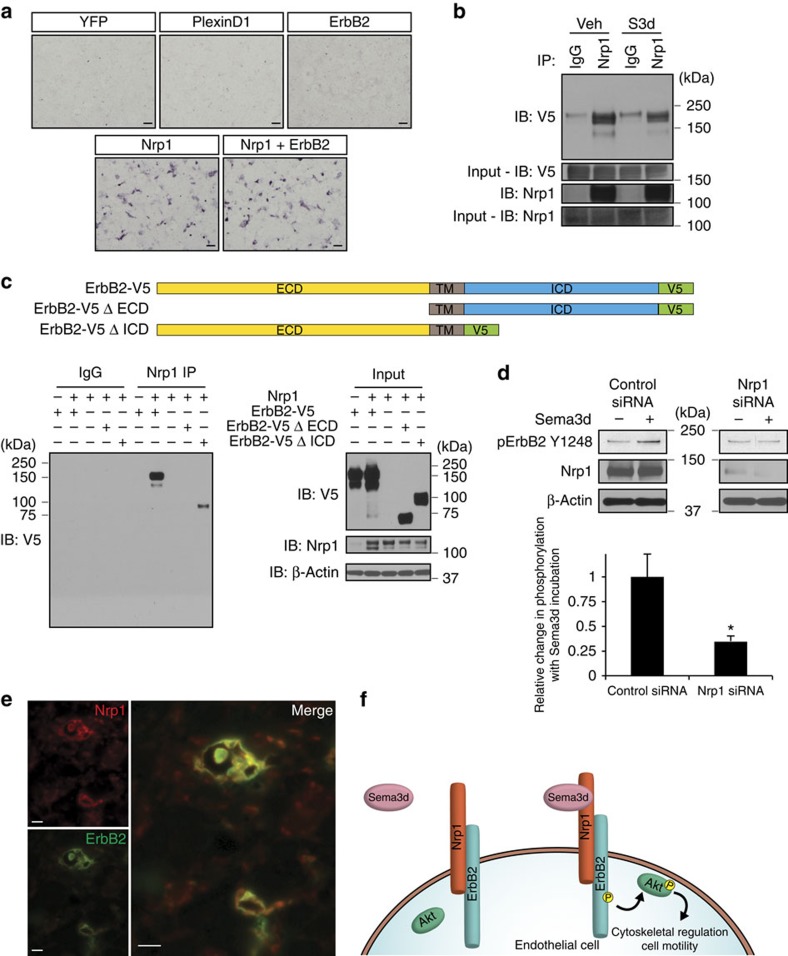
ErbB2 and Nrp1 interact to form a Sema3d receptor. (**a**) Representative photomicrographs of Cos-7 cells expressing indicated receptors after binding and enzymatic detection of alkaline phosphatase tagged Sema3d protein. (**b**) Co-immunoprecipitation of Nrp1 and ErbB2 in the presence or absence of Sema3d (10 nM). (**c**) Graphical representation of ErbB2 deletion constructs (top). Co-immunoprecipitation of Nrp1 and either full length or truncated ErbB2. (**d**) Western blot and quantification for phospho-ErbB2 (Y1248) from HUVECs treated with a Nrp1 or control siRNA, with or without Sema3d for 5 min. *P<0.05 (Student's *t*-test, *n*=4). (**e**) Immunofluorescence of E15.5 hearts shows that ErbB2 (green) and Nrp1 (red) co-localize in coronary vessels. (**f**) Model for Sema3d signalling to coronary endothelial cells. Sema3d binds to the Nrp1 subunit of a Nrp1/ErbB2 complex on endothelial cells. This results in the phosphorylation and activation of ErbB2 and subsequent downstream signalling, including phosphorylation of Akt. ECD, extracellular domain; ICD, intracellular domain; TM, transmembrane domain. Scale bars: **a**,100 μm; **e**, 10 μm.

To test whether Sema3d is capable of directly binding to ErbB2, we performed binding experiments using an alkaline phosphatase (AP)-tagged Sema3d (Sema3d-AP). Sema3d-AP does not bind to Cos-7 cells expressing ErbB2, but does bind to Nrp1-expressing cells (Fig. 5a). Co-expression of ErbB2 with Nrp1 does not increase the binding of Sema3d-AP over that seen with Nrp1 alone, suggesting that Nrp1 may serve to bind Sema3d while ErbB2 may participate in signal transduction (Fig. 5a, Supplementary Fig. 6). Co-immunoprecipitation experiments indicate that ErbB2 and Nrp1 can physically interact in the presence or the absence of Sema3d (Fig. 5b). This interaction is validated by a proximity ligation assay, in which ErbB2 and Nrp1 are shown to be in close physical proximity (<40 nm) when co-transfected in 293T cells (Supplementary Fig. 7). Deletion of the extracellular domain of ErbB2 abolishes the interaction with Nrp1, while deletion of the intracellular domain of ErbB2 does not (Fig. 5c). Serial truncations of the ErbB2 extracellular domain maps the Nrp1 interaction proximal to the transmembrane domain (Supplementary Fig. 8a). Interestingly, Nrp1 interacts with a splice variant of ErbB2 lacking the coding exon 16 upstream of the transmembrane domain (Supplementary Fig. 8b).

siRNA-mediated knockdown of Nrp1 in HUVECs prevents ErbB2 receptor phosphorylation in response to Sema3d (Fig. 5d). In addition, endogenous ErbB2 and Nrp1 co-localize and interact in coronary vessels of the developing heart (Fig. 5e, Supplementary Fig 8c). Taken together, these data support the hypothesis that ErbB2 and Nrp1 heterodimerize to form a functional Sema3d holoreceptor.

## Discussion

In this study, we provide evidence for an unexpected role for ErbB2 in venous endothelial cells where it can partner with Nrp1 to form a receptor for the repellent guidance molecule Sema3d (Fig. 5f). These findings raise the possibility that vascular effects could arise from pharmacologic or other therapeutic approaches targeting ErbB2.

ErbB2 is known to partner with various co-receptors including Egfr, ErbB3 and ErbB4 (ref. 22). In the heart, ErbB2 has been shown to play a critical role in development of the myocardium and has been implicated in control of cardiomyocyte proliferation^23^ and as a potential target for regenerative therapies^24^. Genetic deletion of ErbB2 in cardiomyocytes during development results in dilated cardiomyopathy^18^,^19^. Anticancer drugs targeting ErbB2 can lead to heart failure^25^, presumably through cardiotoxicity. Although the role for the signalling pathways we describe in this report have not been investigated in postnatal animals, we can speculate that the coronary endothelial expression of ErbB2 may provide an alternative mechanism to explain the cardiotoxic effects of such therapies. Perhaps inhibition of ErbB2 in endothelial cells results in endothelial dysfunction and secondary myocardial dysfunction.

Our results indicate that Sema3d is necessary for proper coronary venous connections in the developing heart, but is dispensable for coronary arterial connections. While there is evidence of the molecular determinants of coronary venous origin and development^26^, far less is known about the mechanisms of coronary venous connections. Numerous variations of human coronary vein anatomy have been described^27^,^28^ including anomalous connections to the left atrium^29^ as seen in *Sema3d* null mice. The anatomy of the coronary veins is of clinical significance with regard to cardiovascular interventions such as cardiac pacing, retrograde cardioplegia during cardiopulmonary bypass^30^ and interpretation of cardiac catheterization studies. There has also been recent evidence that the coronary veins instruct sympathetic innervation in the developing heart^31^.

Although our studies have focused on the role of an ErbB2/Nrp1 receptor in venous endothelium, it will be of interest to determine whether this complex plays functional roles in other types of endothelium during development and in the adult, and whether it also functions in other cell types including cancer cells. ErbB2, Nrp1 and semaphorins have all been implicated in various malignancies. ErbB2, also known as Her2, is an important therapeutic target in a subset of breast cancers^32^. ErbB2 overexpression is also seen in gastric and lung cancers^33^,^34^. Similarly, Nrp1 overexpression in breast and lung cancer is associated with worse outcomes^35^,^36^. Furthermore, we analysed gene expression and survival data from the TCGA Research Network (http://cancergenome.nih.gov/) and found ErbB2 and Nrp1 expression positively correlated in lung cancer patients, and those with high ErbB2 and high Nrp1 expression had significantly worse overall survival than those with low levels of both. Multiple classes of semaphorins have been shown to have both pro- and anti-cancer properties and are being investigated as therapeutic targets^37^. The potential role of semaphorin signalling to an ErbB2/Nrp1 receptor in cancer cells and tumour vasculature will be an interesting focus of future studies.

## Methods

### Mice

All mice were maintained on a mixed genetic background. *Sema3d*^*GFPCre*^, *Tie2-Cre,* and *R26*^*Tomato*^ alleles have been previously described^11^,^38^,^39^. *Ephb4*^*LacZ/+*^ and *ephrin-B2*^*LacZ/+*^ mice were obtained from the laboratory of Yoh-suke Mukouyama (NIH). *ErbB2*^*fl/LacZ*^ hearts were obtained from the laboratory of Dr Eldad Tzahor^23^,^40^ (Weizmann Institute of science). All animal protocols were approved by the University of Pennsylvania Institutional Animal Care and Use Committee (IACUC).

### Reagents and antibodies

Human recombinant Sema3e (#3239-S3-025), and human recombinant Sema3d were obtained from R&D Systems. Anti-phospho-Akt (#4060), anti-Akt (#9272), anti-β-actin (#4967), anti-ErbB2 (#4290), anti-ErbB2 (#2165), and anti-phospho-ErbB2 (#2247) were purchased from Cell Signaling, along with anti-Nrp (Santa Cruz, #7239), anti-Nrp (Abcam, #81321), anti-V5 (Life technologies, #R960), anti-ENOS (BD Biosciences, #610296) and anti-GFP (Abcam, #AB6673). Lipofectamine 2000 (Invitrogen) was used as the transfection reagent for HEK293T and Cos-7 cells. Lipofectamine RNAiMAX (Invitrogen, 13778030) was used for siRNA transfections.

### Histology and immunohistochemistry

Samples were harvested, fixed overnight in 2–4% paraformaldehyde and dehydrated through an ethanol series. All samples were paraffin embedded and sectioned. Antibodies used for immunofluorescence were anti-ErbB2 (Cell Signaling, 1:50), anti-phoshpo-ErbB2 Y1248 (Cell Signaling, 1:50), anti-Nrp1 (Santa Cruz, 1:25), anti-GFP (Abcam, 1:250), and anti-eNOS (BD Bioscieznces, 1:250). Hematoxylin and eosin staining was completed using a standard protocol.

For whole-mount X-gal staining, adult and embryonic hearts were isolated and fixed with 2% paraformaldehyde in PBS for 20 min at 4 °C. Following fixation, the samples were washed twice for 10 min in PBS at 4 °C. Embryos were stained in with PBS containing 1 mg ml^−1^ X-gal, 5 mM K_3_Fe(CN)_6_, 5 mM K_4_Fe(CN)_6_, 2 mM MgCl_2_, 0.01% NP-40, 0.01% sodium deoxycholate and incubated overnight at 37 °C.

### Cell culture

Cos-7 and HEK293T cells (originally from ATCC) were cultured in Dubecco's modified Eagle's media (DMEM) from Sigma with 10% fetal bovine serum. Primary HUVECs (Lifeline Cell Technology, lot #1023) were cultured in human endothelial cell culture media, VascuLife EnGS (Lifeline Cell Technology, #LL-0002) (basal EnGS media, 0.2% EnGS, 5 ng ml^−1^ rh EGF, 50 μg ml^−1^ ascorbic acid, 10 mM L-glutamine, 1.0 μg ml^−1^ hydrocortisone hemisuccinate, 0.75 U ml^−1^ heparin sulfate and 2% FBS).

### Microfil

Mice were sacrificed at 2–3 weeks of age to evaluate the coronary artery and vein morphology. Mice were euthanized by CO_2_ inhalation, and the thoracic cavity was opened surgically. The large branches from the aorta were ligated. The vasculature was flushed with normal saline containing heparin (200 U ml^−1^) via a needle inserted into the descending aorta until heart became visibly blanched. The heart was then pressure-fixed with 2% paraformaldehyde. Paraformaldehyde was flushed from the heart in heparinized saline, and coronary vasculature was injected with a radiopaque silicone rubber compound (Microfil MV-122; Flow Tech, Carver, MA) solution prepared in a volume ratio of 1:1 of Microfil diluent with 5% curing agent. Once filling is complete, to prevent the Microfil leakage from the coronary vessels, the accessible major vascular exit points were ligated immediately after filling. Heart was stored at 4 °C for contrast agent polymerization.

### Transwell migration assay

Triplicate transwell inserts (BD Biosciences, 353097) were coated on the underside with 10 μg/ml fibronectin (Roche, 11051407001) and placed in individual wells of a 24-well plate containing either 10 nM recombinant Sema3d or vehicle (PBS) in DMEM. Endothelial cells treated with either an ErbB2 or control siRNA were trypsinized, re-suspended in DMEM containing 0.2% BSA (Gemini, 700-101P) Cells were counted and 10^5^ cells were seeded in each insert and allowed to migrate for 5 h. The migrated cells were fixed in 4% paraformaldehyde for 2 min, permeabilized in methanol for 20 min, and Giemsa (Sigma, GS-500) stained for 25 min. Cell that did not migrate were cleaned out from the inside of the insert with a cotton swab. Three high-power fields of each insert were imaged using an Olympus MVX10 microscope and quantified using ImageJ.

### AP-binding assay

AP-tagged Sema3d-AP (APtag5-Sema3d) was transfected into HEK293T cells and conditioned media was harvested at 48 h. Cos-7 cells were transfected with either YFP, plexin D1, Nrp1, or ErbB2 for 48 h. Transfected cells were incubated with Sema3d-AP conditioned media for 90 min at room temperature. Cell were washed 3 times in PBS and fixed with 4% paraformaldehyde for 20 min at 4 °C. Endogenous AP was inactivated by heating at 65 °C for 2 h. AP activity was detected by incubation with AP buffer (100 mM Tris (pH 9.5), 100 mM NaCl, 50 mM MgCl_2_, 0.33 mg ml^−1^ nitroblue tetrazolium, and 0.05 mg ml^−1^ BCIP) for 1 h at room temperature and recorded on an Olympus MVX10 microscope. Photomicrographs were analysed and quantified using ImageJ software.

### Immunoprecipitation and Western blotting

Immunoprecipitation was performed from either fresh embryonic heart tissue or by transfecting HEK293T cells with Nrp1 and/or ErbB2-V5, or truncated ErbB2 constructs for 48 h. Heart tissue or transfected cells were lysed with immunoprecipitation (IP) lysis buffer (50 mM Tris-HCl; 150 mM NaCl, 1% Ipegal (v/v)), and sonicated on ice. The cell lysates were precleared with protein-G dynabeads (Life technologies, #10004D) for 1 h at 4 °C. Precleared transfected cell lysates were incubated with either 2 μg of Nrp1 antibody, or 2 μg of normal goat IgG (Santa Cruz, #2028) for 2 h at 4 °C. Precleared heart tissue lysates were incubated with either ErbB2 antibody (Cell Signaling #2165, 1:100; 0.945 μg) or 0.945 μg of normal rabbit IgG (Santa Cruz, #2027) for 2 h at 4 °C. Protein-G dynabeads were added and incubaterd overnight at 4 °C. Beads were washed three times in IP wash solution (25 mM Tris-HCl; 150 mM NaCl), eluted in sample buffer (Life technologies, #NP0007; 100 nM DTT, 10% 2-mercaptoethanol), and run on a 4–12% Bis-Tris gradient gel (Life technologies, #NP0336). Gels were transferred to PVDF (Life technologies, #LC2002) and blocked according to antibody manufacturer's instructions.

Blots were probed with anti-ErbB2 (1:1,000), anti-phospho-ErbB2 (1:1,000) anti-phospho-Akt (1:2,000), anti-Akt (1:1,000), anti-β-actin (1:1,000), anti-V5 (1:5,000), or anti-Nrp1 (1:1,000) as per manufacturer's instructions. Visualization was achieved using ECL Prime (GE Life Sciences, #RPN2232) exposed to autoradiography film (Denville, #E3018). Quantification of individual band intensity was performed using ImageJ. Uncropped scans of western blots can be found in Supplementary Fig. 9.

### ErbB2 truncations

Truncations were made to the plasmid ErbB2-pEF-Dest51 using site directed mutagenesis PCR. PfuUltra high-fidelity DNA polymerase (Agilent, 600380) was used to amplify a plasmid with 5′ phosphorylated primers flanking the regions to be deleted Primers are as follows:

ErbB2-V5 Δ ECD

F—5′-GCCGAGCAGAGAGCCAGCCCTCTG-3′

R—5′-GCGGCACAAGGCCGCCAGCTCCAT-3′

ErbB2-V5 Δ ICD

F—5′-TACCTGGGTCTGGACGTGCCAGTG-3′

R—5′-CTTCCGGATCTTCTGCTGCCGTCGCTT-3′

ErbB2-V5 T1

F—5′-CACAAGAACAACCAGCTGGCTCTC-3′

R—5′-GCGGCACAAGGCCGCCAGCTCCAT-3′

ErbB2-V5 T2

F—5′-GGTCTGGGCATGGAGCACTTGCGA-3′

R—5′-GCGGCACAAGGCCGCCAGCTCCAT-3′

ErbB2-V5 T3

F—5′-CGGAACCCGCACCAAGCTCTGCTC-3′

R—5′-GCGGCACAAGGCCGCCAGCTCCAT-3′

ErbB2 Δ16 –V5

F—5′-CCCTCTGACGTCCATCATCTCT-3′

R—5′-GAGTGGGTGCAGTTGATGGGGCAA

Linear PCR products were circularized using T4 DNA ligase (Invitrogen, 15224) and sequenced.

### RNA interference

HUVECs were transfected with Lipofectamine RNAiMAX (Invitrogen, 13778030) using ErbB2 siRNA (Cell Signaling), Nrp1 pre-designed siRNA (Ambion, #4914) or Silencer Negative control siRNA #1 (Ambion, AM4611) in antibiotic-free media. The media was changed to complete HUVEC media after 24 h, and the cells were used at 48 h.

### Proximity ligation assay

HEK293T cells were grown in chamber slides (BD Falcon, Franklin Lakes, NJ) and transfected with 40 ng of cDNA for ErbB2-V5 and/or WT Nrp1. All wells were incubated with a combination of anti-V5 (1:200) and anti-Nrp1 (1:200) primary antibodies for 1 h. Fluorescent visualization of interactions was performed using the Duolink *In Situ* Detection Reagents- Red kit (Sigma-Aldrich, St Louis, MO) following manufacturer's instructions. The Duolink mounting media used includes 4,6-diamidino-2-phenylindole nuclear stain.

### Receptor tyrosine kinase array

Receptor screening was performed using Proteome Profiler Human Phospho-RTK Array Kit (R&D Systems, ARY001B) per manufacturer's instructions. Briefly, HUVECs were incubated with either 10 nM Sema3d or a PBS vehicle control for 30 min. Cells were washed and lysates were made using included lysis buffer plus protease and phosphatase inhibitors. The provided membranes were incubated with Sema3d treated and control cell lysates overnight at 4 °C and probed with the provided Anti-Phospho-Tyrosine-HRP Detection Antibody. Membranes were incubated with a provided chemiluminescent reagent and exposed to autoradiography film (Denville, #E3018). Quantification of dot intensity was performed with ImageJ software.

### Statistics

All data are represented as the mean±s.e.m. Two sets of data were compared using Student's *t*-test. Differences between groups were compared with one-way analysis of variance. Significant analysis of variance results were further analysed by Tukey's multiple comparisons test. *P<0.05, **P<0.01, ***P<0.001, ns, not significant.

### Data availability

The data that support the findings of this study are available from the corresponding author on request.

## Additional information

**How to cite this article:** Aghajanian, H. *et al.* Coronary vasculature patterning requires a novel endothelial ErbB2 holoreceptor. *Nat. Commun.* 7:12038 doi: 10.1038/ncomms12038 (2016).

## Supplementary Material

Supplementary Information Supplementary Figures 1-9

Supplementary Movie 1 Video showing serial H&E cross-sections of a postnatal ErbB2fl/-;Tie2-Cre heart. The pulmonary veins (PV) abnormally connect (green arrowhead) to the right atrium. RA = Right atrium, LA = Left atrium, RV = Right ventricle, LV = Left ventricle, AO = Aorta, PA = Pulmonary artery, CS = Coronary sinus.
