# Supplementary material for: Coronary vasculature patterning requires a novel endothelial ErbB2 holoreceptor
Source: Nat Commun. 2016 Jun 30;7:12038. doi: 10.1038/ncomms12038 (PMC4931334; doi:10.1038/ncomms12038)
Supplement: Supplementary Information — Supplementary Figures 1-9 [file ncomms12038-s1.pdf]

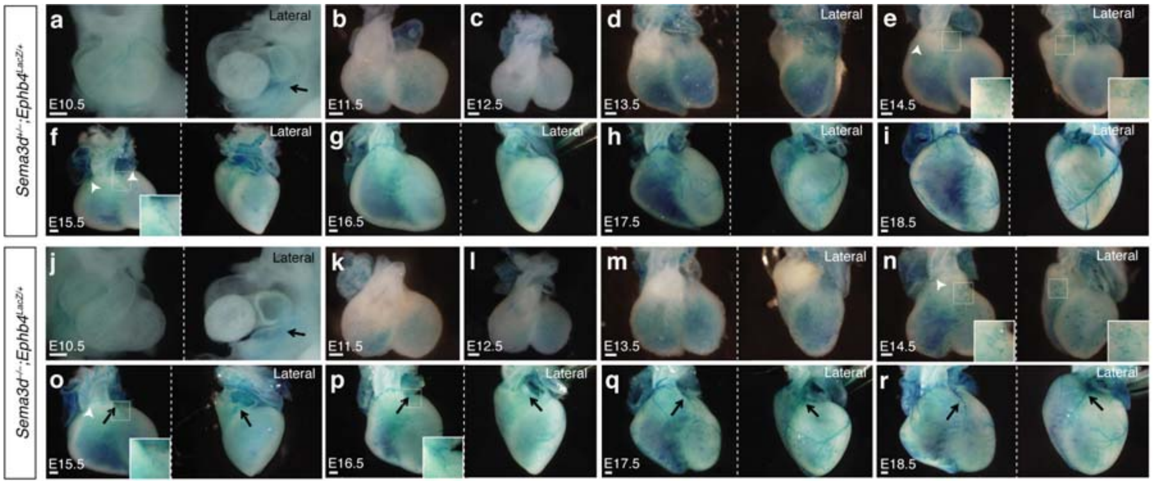

Supplementary Figure 1

(**a-r**) Whole mount X-gal staining on a developmental time-course of hearts from *Sema3d*$^{+/-}$;*Ephb4*$^{LacZ/+}$ and *Sema3d*$^{-/-}$;*Ephb4*$^{LacZ/+}$ embryos. In both the Sema3d nulls and controls, Ephb4 expression can be seen as early as E10.5 in the sinus venosus (**a, j; arrows**). Ephb4 positive endothelial cells are not seen (**b-d, k-m**) outside of the immediate area surrounding the sinus venosus until E14.5 when the subepicardial endothelial cells re-specify to a venous identity, and are seen in both the Sema3d null and control hearts beginning at E14.5 (**e, n; inset, arrowheads**). By E15.5 more mature venous vessels are present in the control and *Sema3d* null hearts (**f, o; inset, arrowheads**), and can be seen near the left atrium (**o; arrow, inset**). One day later (E16.5), the anomalous connections of the coronary veins are grossly distinguishable in the nulls (**p; arrow, inset**) and continue to develop through gestation (**q, r; arrows**), but are not seen in the controls (**g-i**). These data indicate that early in development Sema3d null venous endothelial cells migrate and pattern appropriately but by E15.5 incorrect venous connections begin to form. Scale bars = 100μm.

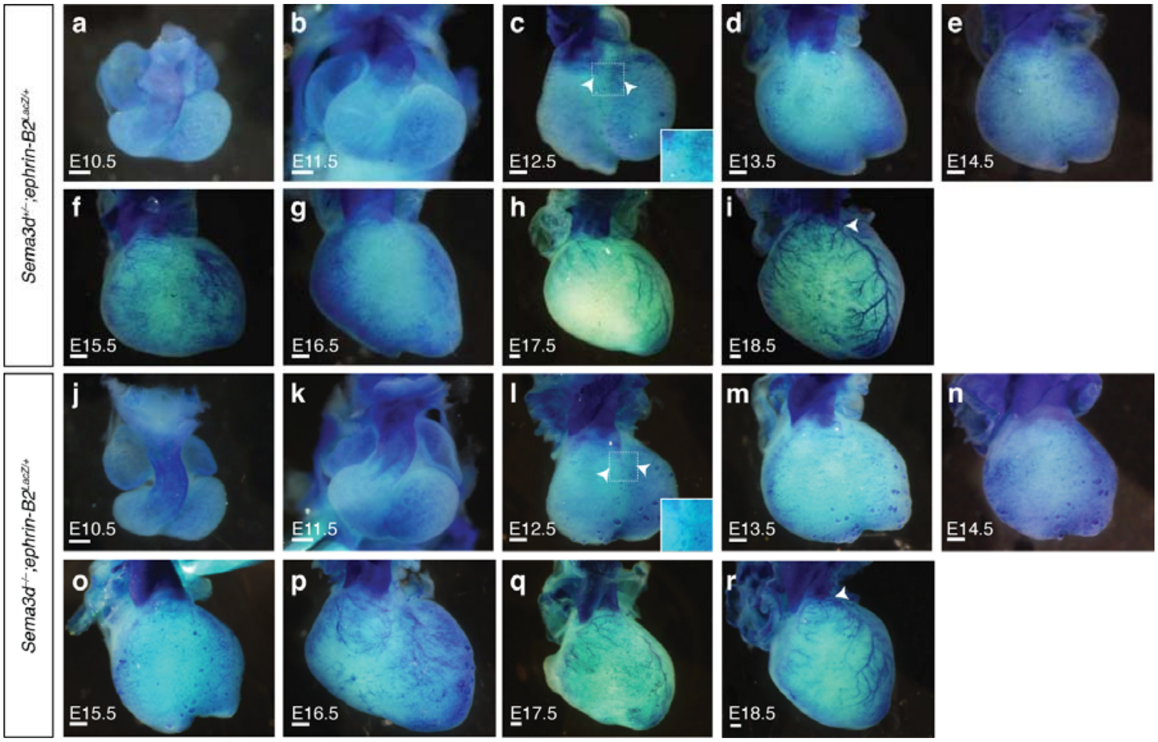

Supplementary Figure 2

(**a-r**) Whole mount X-gal staining on a developmental time-course of hearts from *Sema3d*$^{+/-}$*;ephrin-B2*$^{LacZ/+}$ and *Sema3d*$^{-/-}$*;ephrin-B2*$^{LacZ/+}$ embryos. Ephrin-B2 positive arterial endothelial cells are not detected (**a-b, j-k**) until E12.5 when a peritruncal vascular plexus is seen (**c, l; arrowheads, insets**) in the *Sema3d* control and null hearts. Arterial development continues normally in the *Sema3d* null and control hearts (**d-h, m-q**) including the origins of the coronary arteries (**i, r**) at E18.5. Scale bars = 100µm.

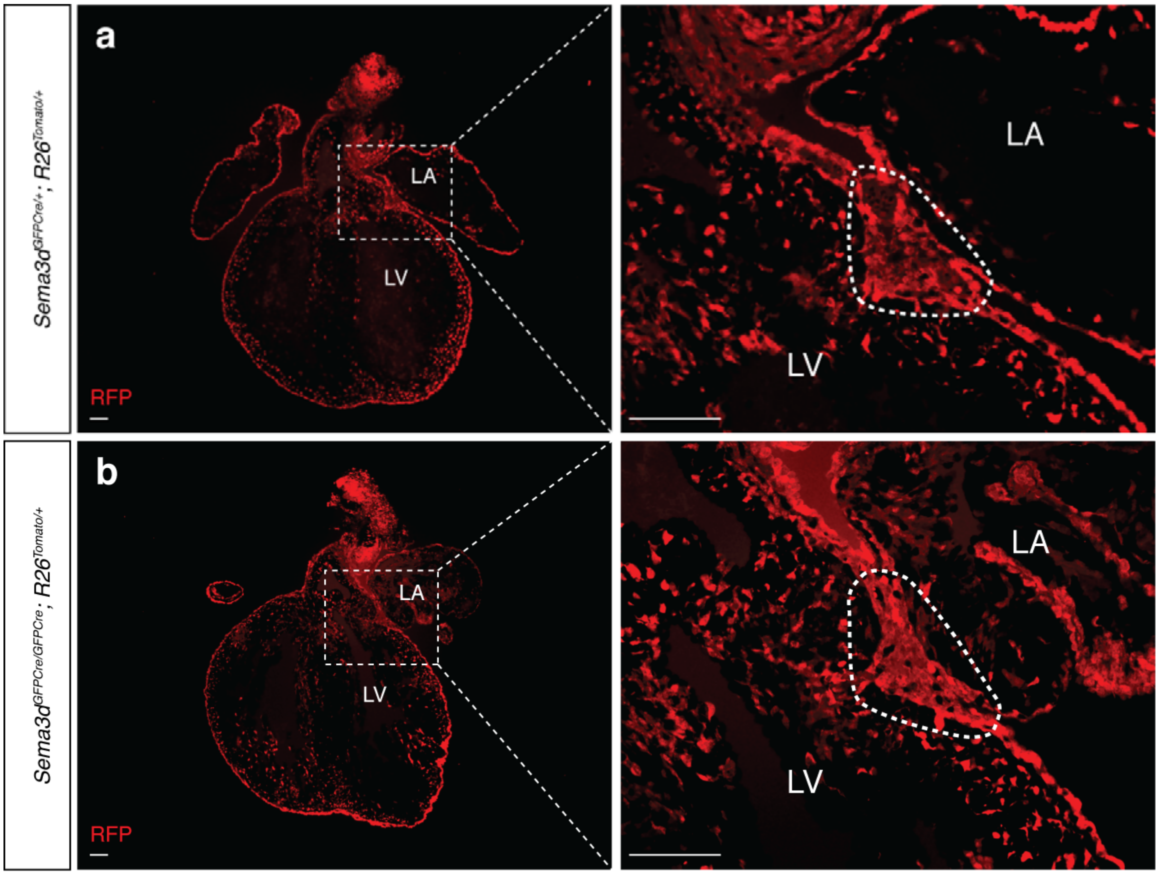

Supplementary Figure 3

(**a, b**) Immunofluorescence for RFP (red) on frontal sections of E14.5 $Sema3d^{GFPCre/+}$; $R26^{Tomato/+}$ (**a**) and $Sema3d^{GFPCre/GFPCre}$; $R26^{Tomato/+}$ (**b**) hearts. Sema3d expressing cells similarly fatemap to the left anterior AV groove in both the control (a**, outline**) and null (**b, outline**) hearts. LA = Left atrium, LV = Left ventricle. Scale bars = 100µm.

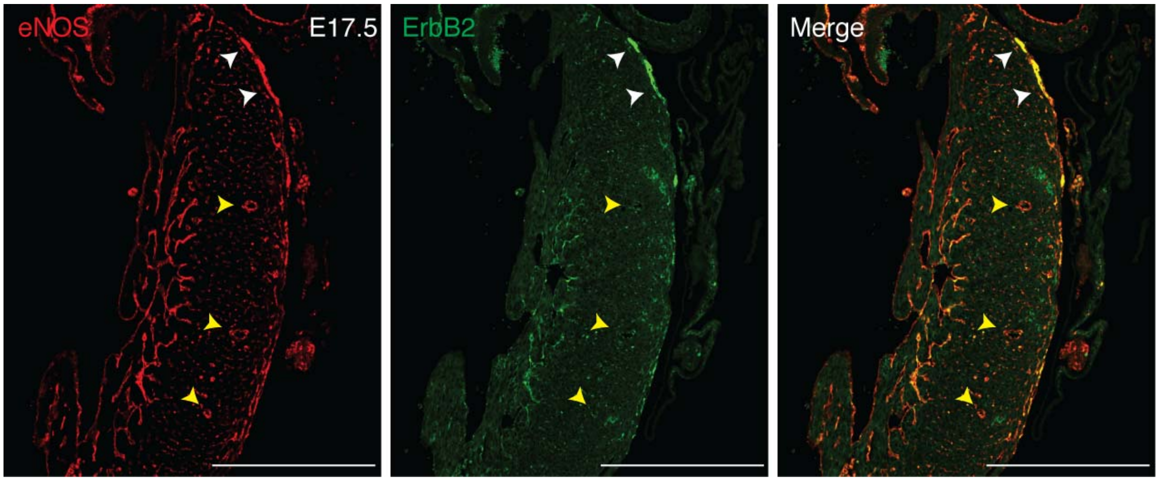

Supplementary Figure 4

Immunofluorescence for the endothelial marker eNOS (red) and ErbB2 (green) on frontal sections from E17.5 heats showing the left ventricular wall. Subepicardial vessels (venous) are ErbB2 positive (yellow arrowheads), while deeper, myocardia vessels (arterial) are negative for ErbB2. Scale bars = 100µm.

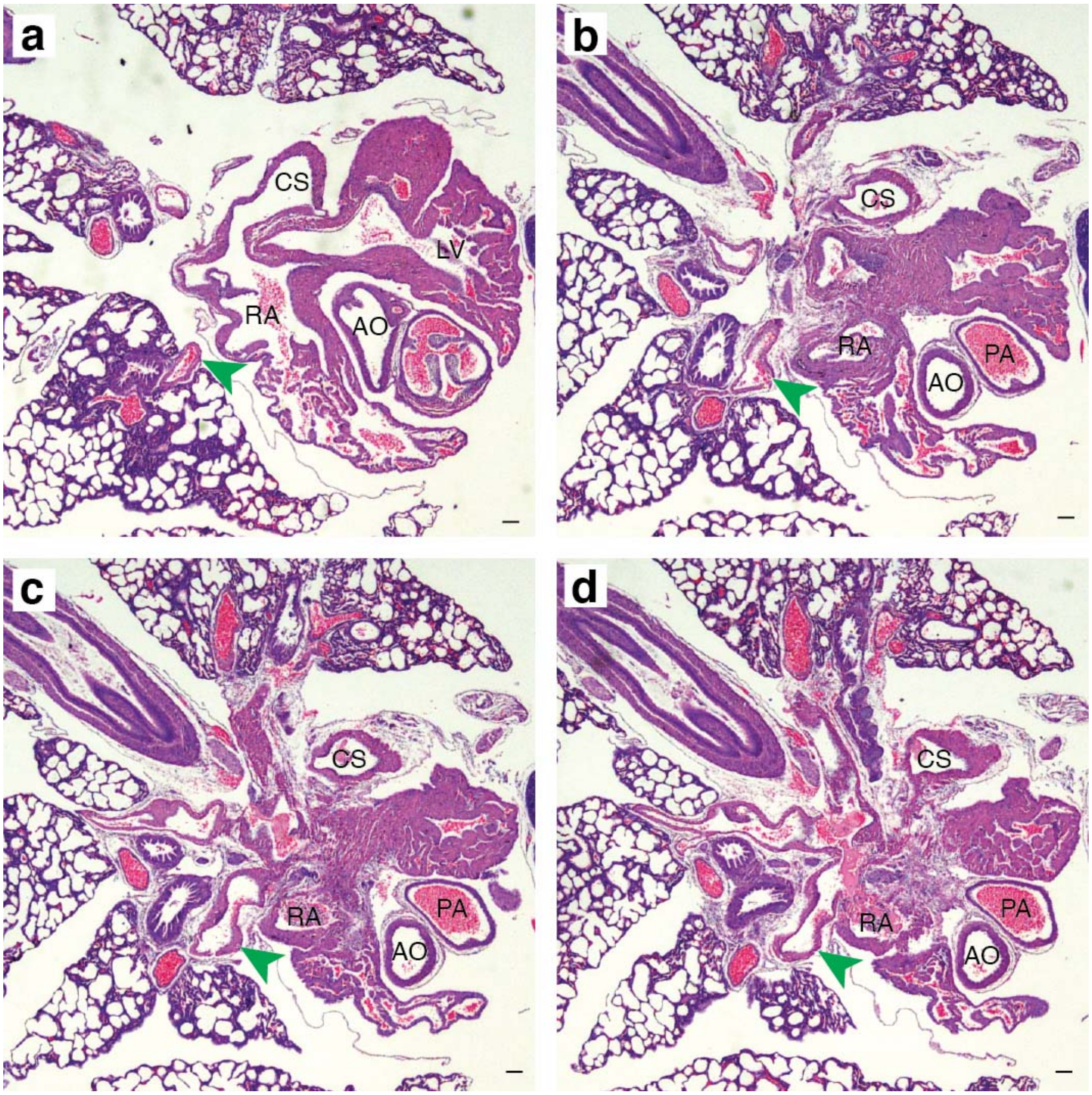

Supplementary Figure 5

(**a-d**) H&E stained cross sections of a postnatal *$ErbB2^{fl/-}$;Tie2-Cre* heart. The pulmonary veins (green arrowhead) abnormally connect to the right atrium (RA) (**d**) resulting in an anomalous pulmonary connection (APVC). RA = Right atrium, LA = Left atrium, RV = Right ventricle, LV = Left ventricle, AO = Aorta, PA = Pulmonary artery, CS = Coronary sinus. Scale bars = 100μm.

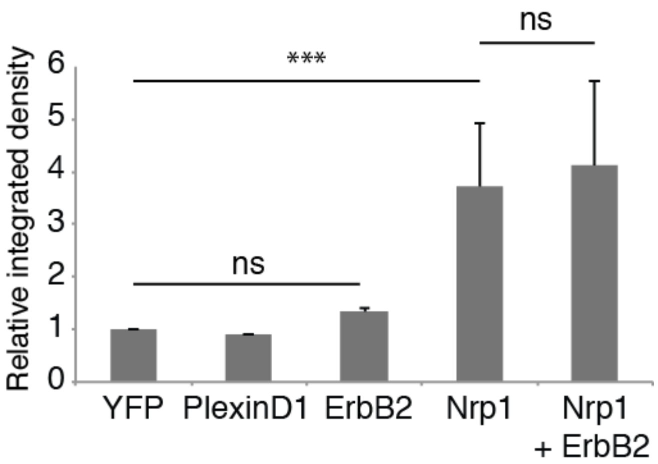

Supplementary Figure 6

Quantification of the relative integrated density of Cos-7 cells expressing either a YPF control, plexin D1, ErbB2, Nrp1, or ErbB2 and Nrp1 bound with alkaline phosphatase (AP) tagged Sema3d (Sema3d-AP) and developed using an AP colorimetric assay. ***$P < 0.001$, ns = not significant. (one-way ANOVA between groups $P < 0.001$; post-hoc multiple comparisons, Tukey’s test)

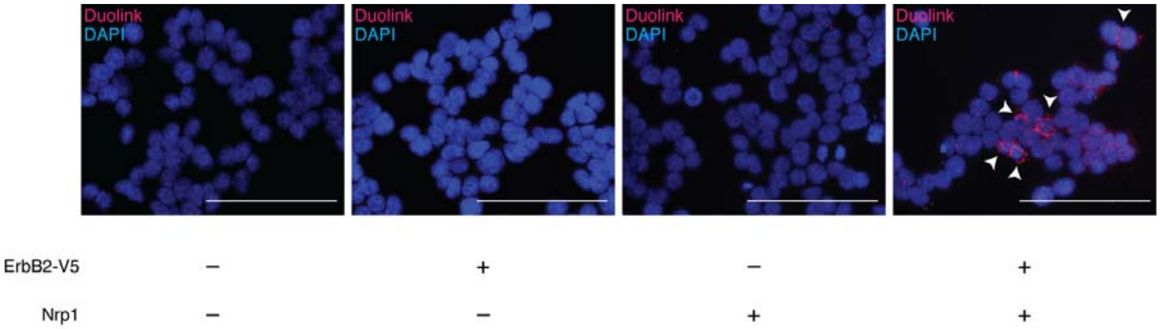

Supplementary Figure 7

Duolink proximity ligation assay (PLA) of 293T cells transfected with ErbB2-V5, Nrp1, or both. Red signal indicates physical proximity of proteins (<40 nm) and can be seen in the cells that were co-transfected with ErbB2-V5 and Nrp1 (**arrowheads**), but not in the controls. All conditions were incubated with anti-V5 and anti-Nrp1 antibodies. Scale bars = 100μm.

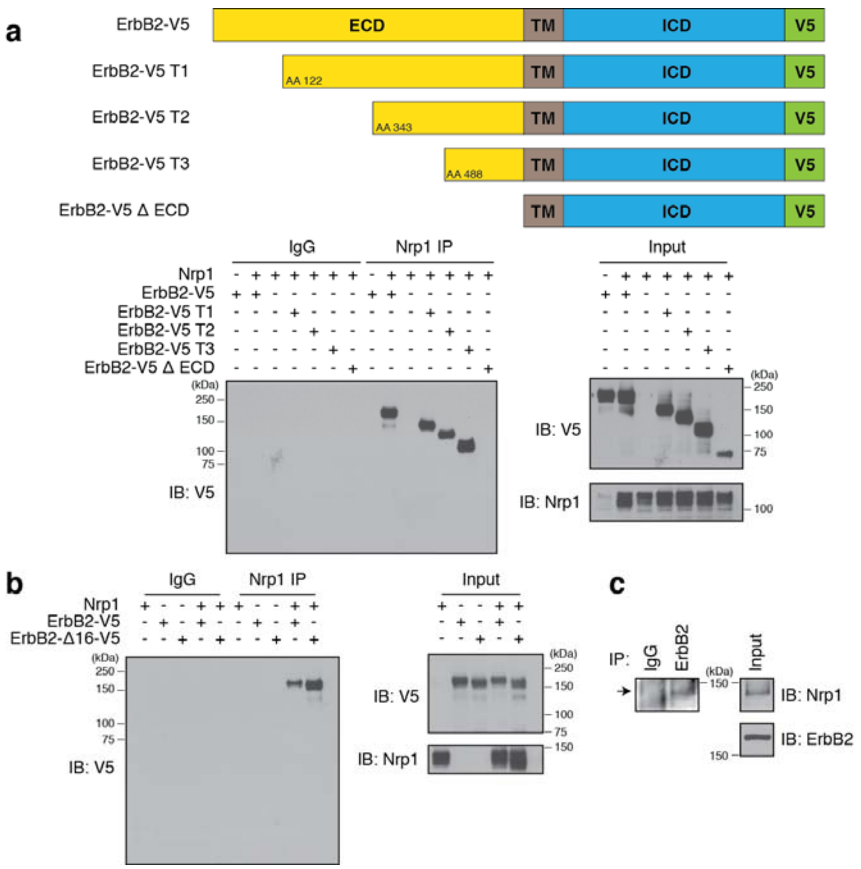

Supplementary Figure 8

(**a**) Graphical representation of ErbB2 truncations (top). Co-immunoprecipitation of Nrp1 with either a full length (ErbB2-V5), progressive ErbB2 extracellular truncation (T1-T3), or complete extracellular truncation (ErbB2-V5 ΔECD) protein. The shortest truncation of ErbB2 (T3) still interacts with Nrp1, while a complete loss of the extracellular domain completely abrogates this interaction. (**b**) Co-immunoprecipitation of Nrp1 with either full length ErbB2 (ErbB2-V5) or a variant of ErbB2 lacking exon 16 (ErbB2-Δ16-V5). (**c**) Co-immunoprecipitation of endogenous ErbB2 and Nrp1 from lysates derived from ~30 isolated whole E14.5 WT hearts.

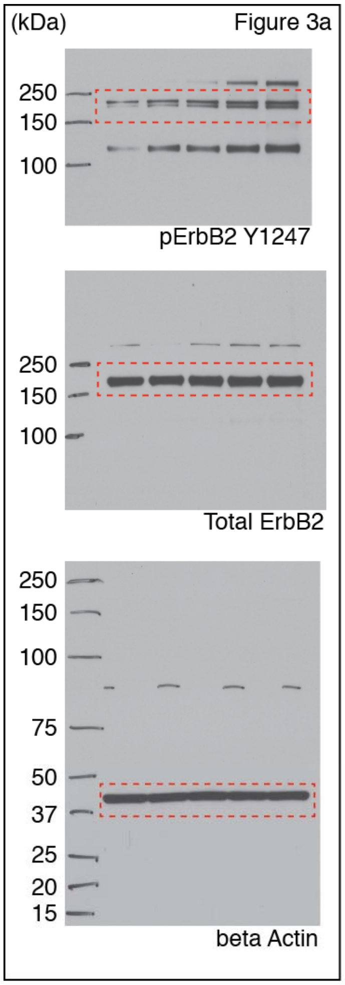
(kDa)
Figure 3a
250
150
100
pErbB2 Y1247
250
150
100
Total ErbB2
250
150
100
75
50
37
25
20
15
beta Actin

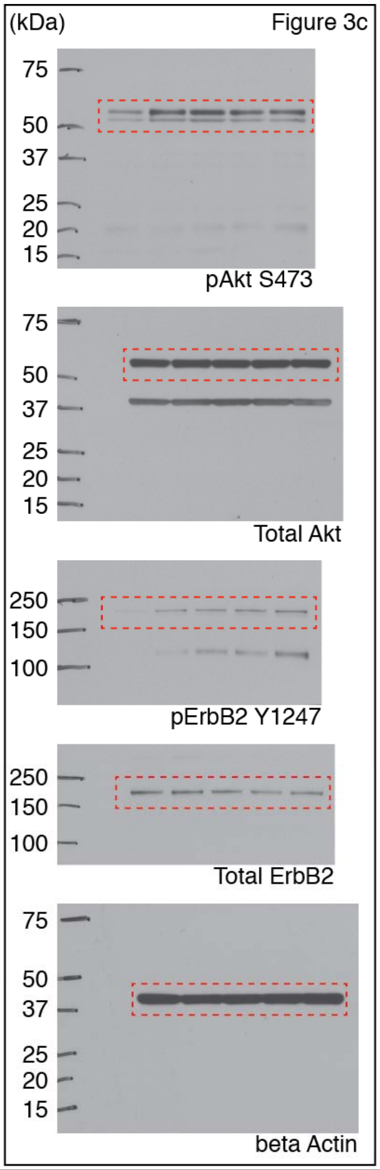
(kDa)
Figure 3c
75
50
37
25
20
15
pAkt S473
75
50
37
25
20
15
Total Akt
250
150
100
pErbB2 Y1247
250
150
100
Total ErbB2
75
50
37
25
20
15
beta Actin

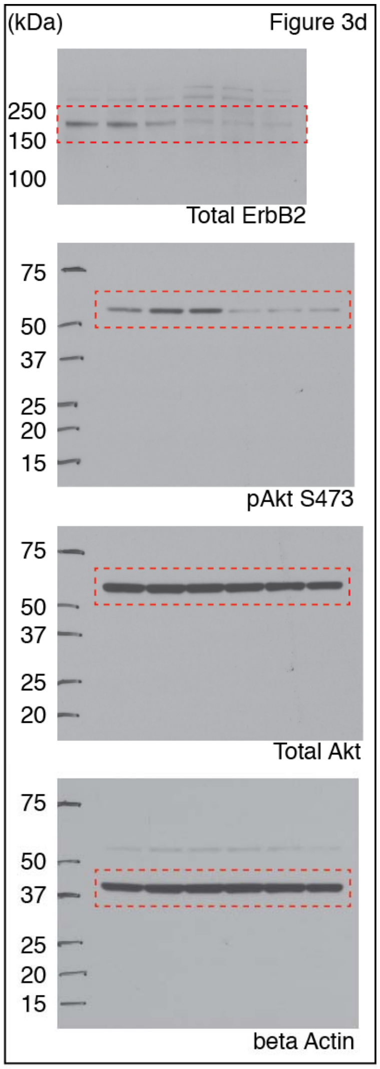
(kDa)
Figure 3d
250
150
100
Total ErbB2
75
50
37
25
20
15
pAkt S473
75
50
37
25
20
Total Akt
75
50
37
25
20
15
beta Actin

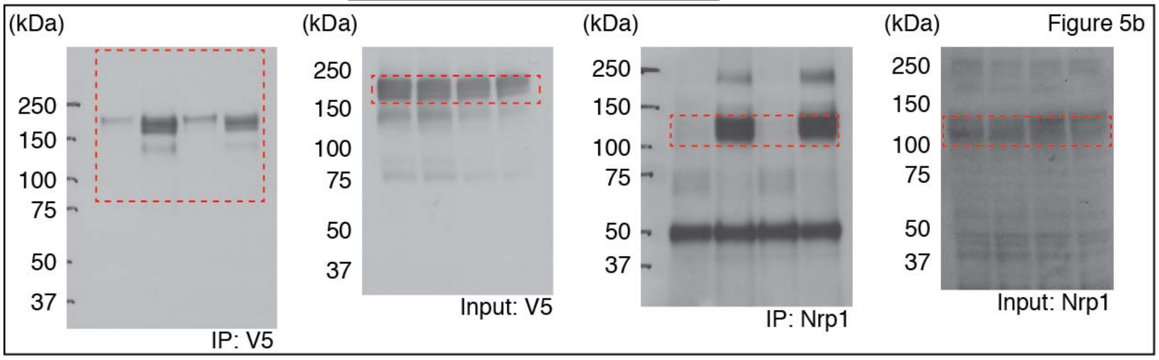
(kDa)
250
150
100
75
50
37
IP: V5
(kDa)
250
150
100
75
50
37
Input: V5
(kDa)
250
150
100
75
50
37
IP: Nrp1
(kDa)
Figure 5b
250
150
100
75
50
37
Input: Nrp1

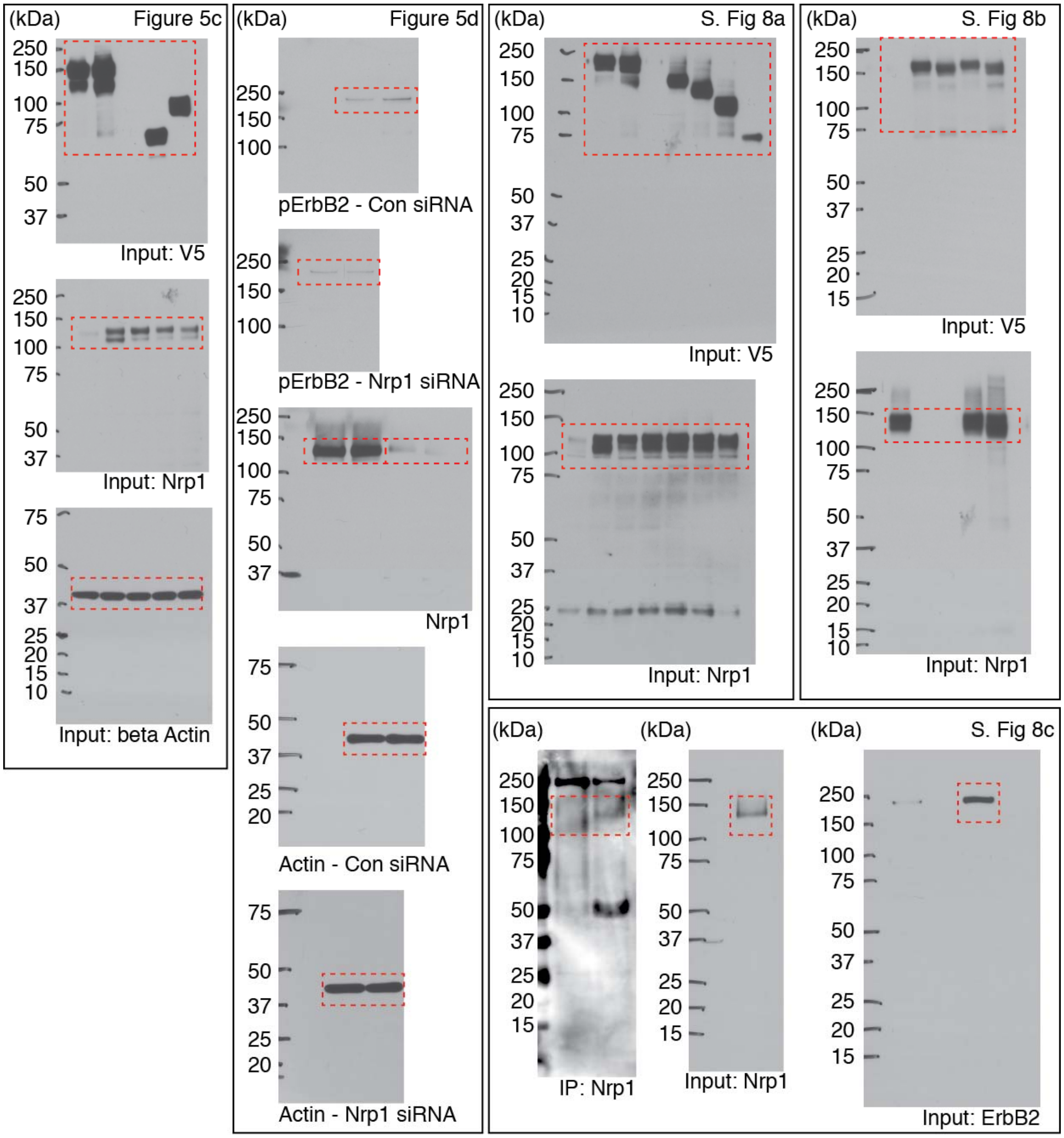

Supplementary Figure 9

Uncropped scans of western blots presented in this manuscript. Red dotted box indicates areas that were cropped for use in figures. Corresponding figure numbers are indicated.
